# Outcomes of Philadelphia Positive Acute Lymphoblastic Leukemia in Adolescent and Young Adults

**DOI:** 10.7759/cureus.32467

**Published:** 2022-12-13

**Authors:** Umair Ahmed, Danyal Ahmed, Munazza N Awan, Usman Ahmad, Bushra Ahsan, Raheel Iftikhar, Muhammad Ayaz Mir, Syed W Bokhari

**Affiliations:** 1 Medical Oncology, Shaukat Khanum Memorial Cancer Hospital and Research Centre, Lahore, PAK; 2 Internal Medicine, Shifa International Hospital, Islamabad, PAK; 3 Hematology and Oncology, Armed Forces Bone Marrow Transplant Centre/National Institute of Blood and Marrow Transplant, Rawalpindi, PAK; 4 Hematology and Oncology, Shifa International Hospital, Islamabad, PAK

**Keywords:** philadelphia chromosome-positive acute lymphoblastic leukemia (ph+ all), low-income countries, allogeneic stem cell transplant (allo-sct), tyrosine kinase inhibitors (tkis) therapy, adolescent and young adult (aya)

## Abstract

Background

Philadelphia chromosome-positive acute lymphoblastic leukemia (Ph+ ALL) accounts for 25% of acute lymphoblastic leukemia cases in the adolescent and young adult (AYA) age subgroup. It is associated with poor outcomes and is considered a standard indication for allogeneic stem cell transplant (Allo-SCT). Improved outcomes have been reported with addition of tyrosine kinase inhibitors (TKIs) to chemotherapy in children and the role of Allo-SCT is now being debated in the first remission. Complete response (CR) at three months is associated with improved survival even without Allo-SCT in first CR. In this study, we have analyzed disease-free survival (DFS), overall survival (OS), and factors affecting survival outcomes of Ph+ ALL in the AYA subgroup, in resource-limited settings treated with chemotherapy and TKIs.

Materials and methods

This is a retrospective, multicenter cohort study of Ph+ ALL AYA patients, aged 18-40 years, and registered between January 2015 and December 2020. Primary objectives are to calculate disease-free survival (DFS) and overall survival (OS). Secondary objectives are to identify prognostic factors affecting response rates and outcomes. List of cases was obtained from hospital information system (HIS) and data were collected from patient case notes and electronic medical records. Data analysis was done utilizing the SPSS statistical program (Armonk, NY: IBM Corp.).

Results

Forty-nine patients were identified with Ph+ ALL with a median age of 23 years (range: 18-40 years) and a male-to-female ratio of 2.5:1. None of the patients had central nervous system (CNS) disease. White cell count was >30,000 per mm^3^ in 26% of patients, while 13% had additional cytogenetic abnormalities. Thirty-three percent patients received adult (hyper-cyclophosphamide, vincristine, Adriamycin, and dexamethasone {CVAD}) protocols while 67% received pediatric-inspired (Berlin-Frankfurt-Munster {BFM} 2000 or UK-ALL 2003/2011) protocols. TKI therapy was received by 66% of patients during treatment (early: 37%; late: 29%) and 34% did not receive TKIs due to financial constraints. CR after induction was achieved in 69% cases. Induction mortality was 16%. The median DFS for the entire cohort was 27 months (0.93-53.06) and the median OS was 29 months (8.89-49.10). The median OS in Allo-SCT group was not reached vs 8.0±8.8 months (p=0.05) with chemotherapy only. The OS was significantly better in patients with no additional cytogenetic abnormalities, pediatric-inspired chemotherapy protocols, early use of TKIs in induction phase, Allo-SCT, and post-Allo-SCT use of TKIs.

Conclusion

Addition of TKIs to pediatric-inspired chemotherapy protocols in Ph+ ALL AYA patients and Allo-SCT results in better overall survival. TKI availability remains a significant issue in low-income countries due to significant financial burden on the patients. Allo-SCT continues to be an attractive option, particularly in low-income countries providing an option for cure in Ph+ ALL.

## Introduction

Philadelphia chromosome-positive acute lymphoblastic leukemia (Ph+ ALL) makes up about 20-30% of all ALL [1]. The prevalence of Ph+ ALL increases with age, ranging from 5% of pediatric ALL to 25% of adult ALL and nearly 50% of elderly patients above 60 years of age [2]. Traditionally, Ph+ ALL has been associated with poor long-term outcomes, with a five-year survival rate of less than 20% [3]. The addition of tyrosine kinase inhibitors (TKIs) to conventional chemotherapy has improved response rates and survival outcomes in both pediatric and adult populations, and allogeneic stem cell transplant (Allo-SCT) is being challenged in first complete remission (CR1), particularly in the pediatric setting [4,5]. This has led to the utilization of a similar approach for adolescents and young adults (AYA) between the ages of 18 and 40 years. Achievement of complete metabolic response at three months is independently associated with improved survival and helps identify patients with excellent long-term outcomes even without stem cell transplantation in CR1 [6]. However, in low-income countries where TKI procurement is prohibitively expensive for most patients, treatment with conventional chemotherapy and limited TKI use followed by allogeneic stem cell transplantation (Allo-SCT) still offer survival but with associated morbidity and mortality [7].

This study presents outcomes of Ph+ ALL AYA patients in a multicenter, retrospective cohort study conducted at the following three tertiary care hematology and transplant centers in Pakistan: Shaukat Khanum Memorial Cancer Hospital, Lahore (data collection and analysis); Shifa International Hospital, Islamabad; and Armed Forces Bone Marrow Transplant Centre/National Institute of Blood and Marrow Transplant (AFBMTC/NIBMT), Rawalpindi, between January 1, 2015, and December 31, 2020. The aim of this study was to assess outcomes in terms of disease-free survival (DFS) and overall survival (OS) with intensive chemotherapy and TKIs, along with the impact of Allo-SCT and other factors on survival outcomes.

## Materials and methods

We retrospectively reviewed the medical records of 66 Ph+ ALL AYA patients. Inclusion criteria were all Ph+ ALL patients, between the age of 18 and 40 years with the availability of minimal essential data required for outcome analysis. Exclusion criteria included Ph+ ALL patients less than 18 years or older than 40 years of age, known chronic myeloid leukemia (CML) patients with lymphoid blast crisis, and those with missing minimal essential data for analysis of survival outcomes. Six patients were excluded for being outside the defined age limit. Eleven patients with CML in lymphoid blast crisis were also excluded, leaving 49 eligible patients who were enrolled in the study. The study was approved by the respective institutional review boards of each institution.

Treatment protocol

Treatment included induction chemotherapy either with adult protocols (hyper-CVAD) or pediatric-inspired ones (UKALL 2003/2011 and Berlin-Frankfurt-Munster {BFM} 2000). TKIs (imatinib 400 mg OD and nilotinib 400 mg BID) were added to the chemotherapy backbone subject to availability. Post-induction assessment included morphology, flow cytometry, cytogenetics, and minimal residual disease (MRD) assessment by flow cytometry. Patients in CR after the induction phase went on to receive consolidation and central nervous system intensification phases of their respective protocols. Patients with an available donor in first complete remission (CR1) were offered Allo-SCT. Patients who were not eligible or able to proceed with Allo-SCT were placed on maintenance chemotherapy with a TKI if available. The follow-up for data analysis was censored on June 30, 2022.

Definitions

Ph+ ALL was defined as ALL carrying the t(9;22) translocation detected by standard karyotyping or fluorescence in situ hybridization (FISH) analysis and/or positivity for BCR-ABL fusion transcript detected by real-time polymerase chain reaction (PCR). CR post-induction was defined as less than 5% blasts in bone marrow with no extramedullary disease and normalization of blood counts or a negative MRD if the assessment for CR was conducted by flow cytometry.

Statistical analysis

Primary study endpoints were DFS defined as the time from diagnosis with ALL until the time of progression of the disease and overall survival (OS) defined as the length of time from diagnosis to death or last follow-up. Categorical variables were reported as total number (n) and percentages. Continuous variables were reported as a median and interquartile range. A two-tailed p≤0.05 indicated statistical significance. Survival curves for OS and DFS were plotted according to the Kaplan-Meier method and compared using the log-rank method. All statistical analyses were performed using SPSS v.21.0 for Windows (Armonk, NY: IBM Corp.).

## Results

Out of 66 Ph+ ALL patients, 49 were eligible for this study. Six patients were excluded for being outside of the defined age limit (16-40 years). Eleven patients with chronic myeloid leukemia (CML) with lymphoid blast crisis were also excluded. The demographics of eligible patients are presented in Table 1. Out of the 49 eligible patients, 35 (71%) were males and 14 (29%) were females, with a median age of 23 years (18-40 years). Thirteen (26%) patients had a white blood cell count >30,000 per mm^3^ defined as a high-risk disease. None of the patients had central nervous system (CNS) involvement at baseline. Forty-three (87%) patients had only Philadelphia chromosome-positive t(9;22) on karyotyping, while six (13%) patients also had additional cytogenetic abnormalities. Eastern Cooperative Oncology Group (ECOG) performance statuses of 0, 1, and 2 were present in 21 (42%), 20 (41%), and eight (17%) patients, respectively.

**Table 1:** Demographics of eligible patients.

Patient characteristics	Enrolled patients (n=49)
Age (years)	
Median	23 (18-40)
Gender, number (%)	
Male	35 (71)
Female	14 (29)
White cell count per mm^3 ^, number (%)	
>30,000	13 (26)
<30,000	36 (74)
Central nervous system disease, number (%)	
Yes	0
No	49 (100)
Karyotyping, number (%)	
t(9;22)	43 (87)
t(9;22) plus additional cytogenetic abnormalities	6 (13)
Eastern cooperative oncology group performance scale, number (%)	
0	21 (42)
1	20 (41)
2	8 (17)

Sixteen (33%) patients received an adult chemotherapy protocol (H-CVAD) with nine (56%) receiving TKIs during treatment. Thirty-three (67%) patients received pediatric-inspired chemotherapy protocols (BFM 2000 or UK-ALL2003) with 23 (69%) receiving TKIs as well during treatment. Overall, 32 (66%) patients received TKIs with chemotherapy and 17 patients (34%) did not receive them, usually due to non-availability and/or financial constraints. In the former patients, TKIs were added to the chemotherapy backbone “early,” i.e., during induction in 18 (37%), and “late,” i.e., after induction in 14 (29%) patients.

**Figure 1:**
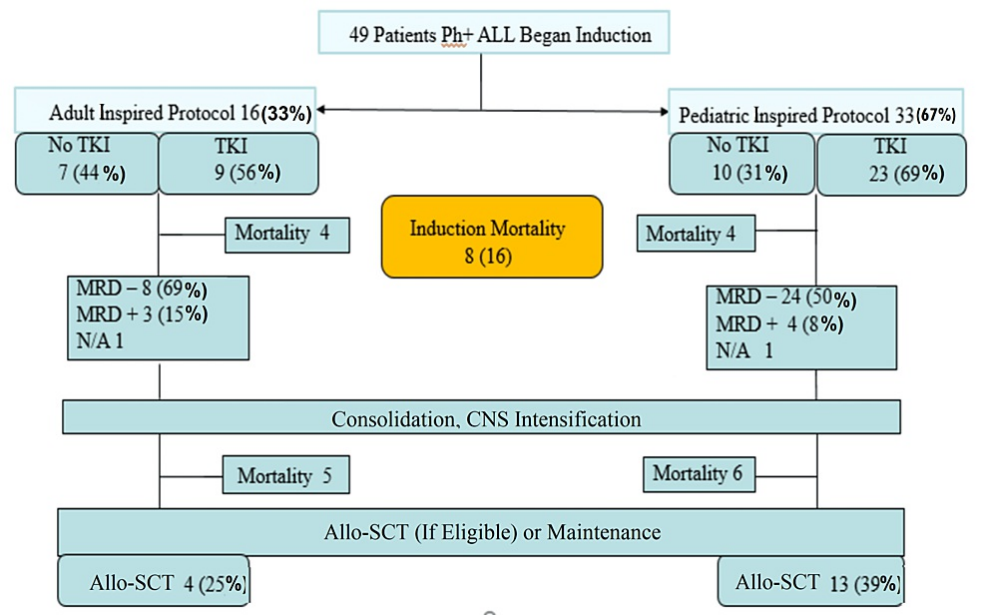
Flow diagram showing treatment outcomes. Allo-SCT: allogeneic stem cell transplant; CNS: central nervous system; MRD: minimal residual disease; N/A: not available; Ph+ ALL: Philadelphia chromosome-positive acute lymphoblastic leukemia; TKI: tyrosine kinase inhibitor

Induction mortality was eight (16%) with four deaths each in the adult and pediatric-inspired chemotherapy groups. The leading cause of mortality was sepsis secondary to Gram-negative bacteremia in four (50%) patients, and fungal pneumonia in four (50%) patients. Patients in the adult chemotherapy group had more profound cytopenia and required frequent dose reductions. No TKI (imatinib and nilotinib) specific complications were noted in any patient. Post-induction, eight out of 12 (69%) surviving patients were MRD negative in the adult chemotherapy group while 24 out of 33 (73%) surviving patients were MRD negative in the pediatric-inspired chemotherapy group. Figure 1 below (the flow diagram) summarizes treatment outcomes.

**Figure 2:**
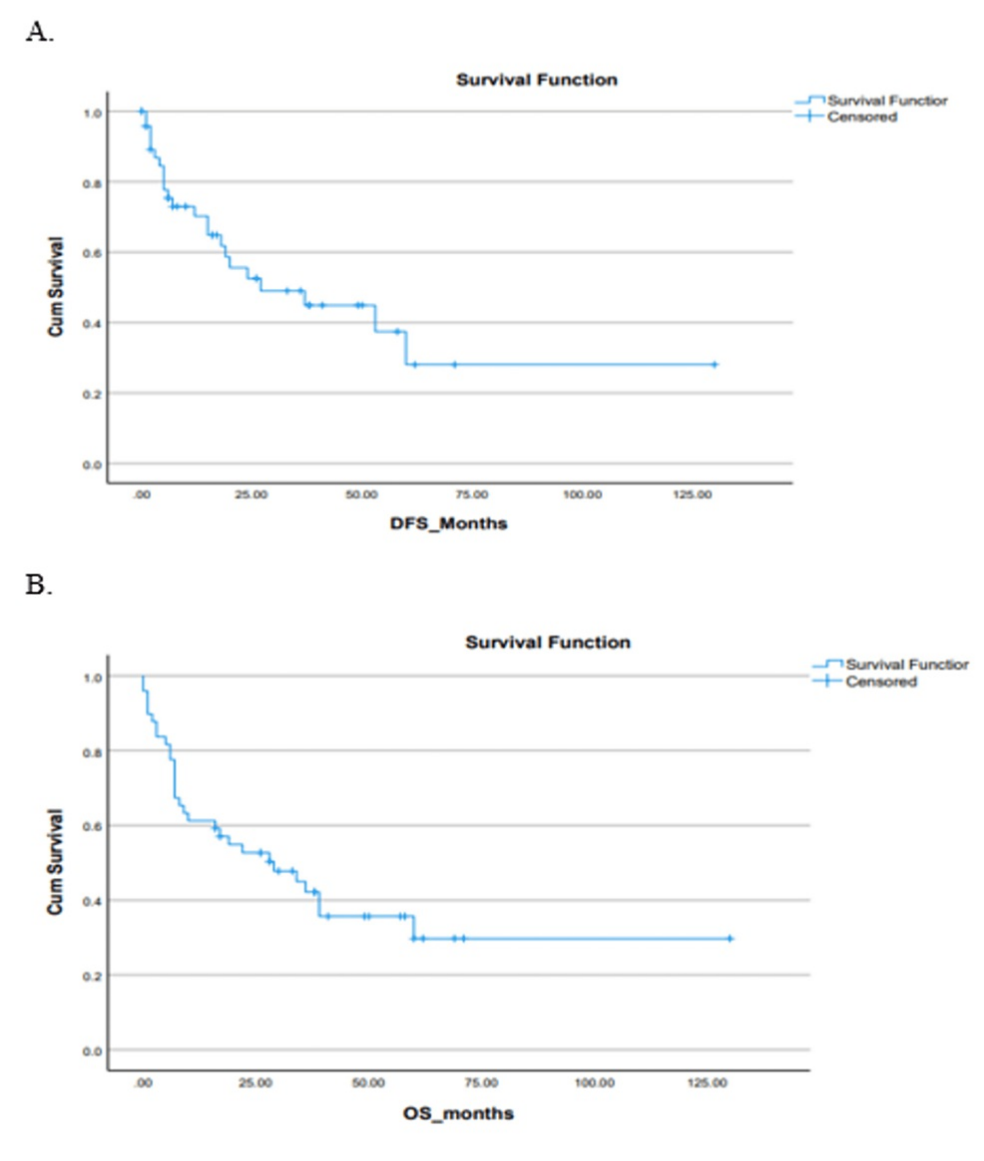
Kaplan-Meier curve depicting (A) the DFS of the cohort and (B) the OS of the cohort. DFS: disease-free survival; OS: overall survival; Cum survival: cumulative survival

Out of the 49 patients, 17 (34%) patients underwent Allo-SCT - 14 (82.4%) in CR1 and three (17.6%) in second complete remission (CR2). All were matched sibling donor Allo-SCTs. Myeloablative conditioning regimens were used in all patients. Only 10 (58%) patients received TKI maintenance after Allo-SCT due to limited access to TKIs. Post-Allo-SCT, nine (53%) patients remained in remission, four (25%) patients relapsed with CNS being the primary site of relapse, and four (24%) patients were lost to follow-up. The median follow-up of the entire cohort was 31 months (3.7-54.8). The median DFS for the entire cohort was 27 (0.9-53.1) months. The median OS was 29 months (8.8-49.1) (Figure 2).

**Figure 3:**
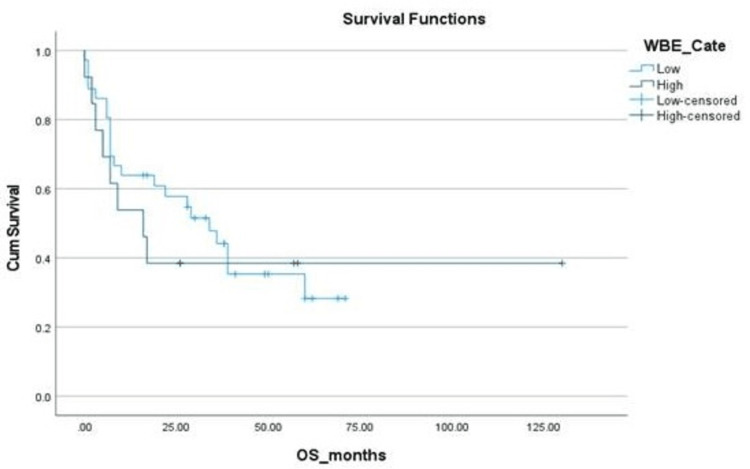
Kaplan-Meier curve depicting OS in relation to the white blood cell category. OS: overall survival; WBC_Cate: white blood category; Cum survival: cumulative survival

**Figure 4:**
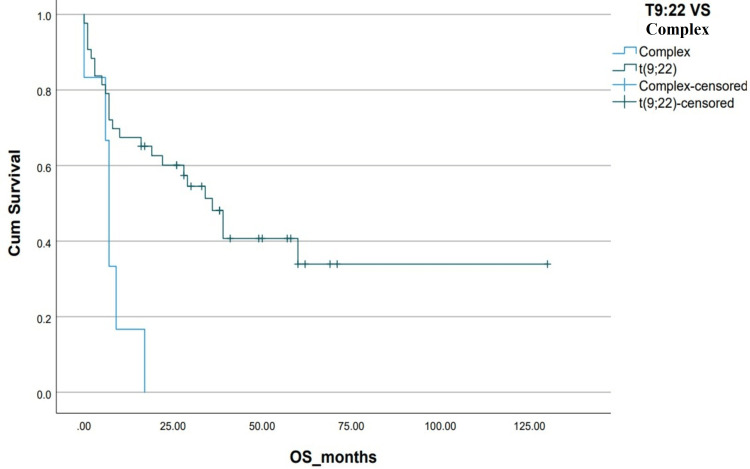
Kaplan-Meier curve depicting OS in relation to cytogenetics. OS: overall survival; Cum survival: cumulative survival

**Figure 5:**
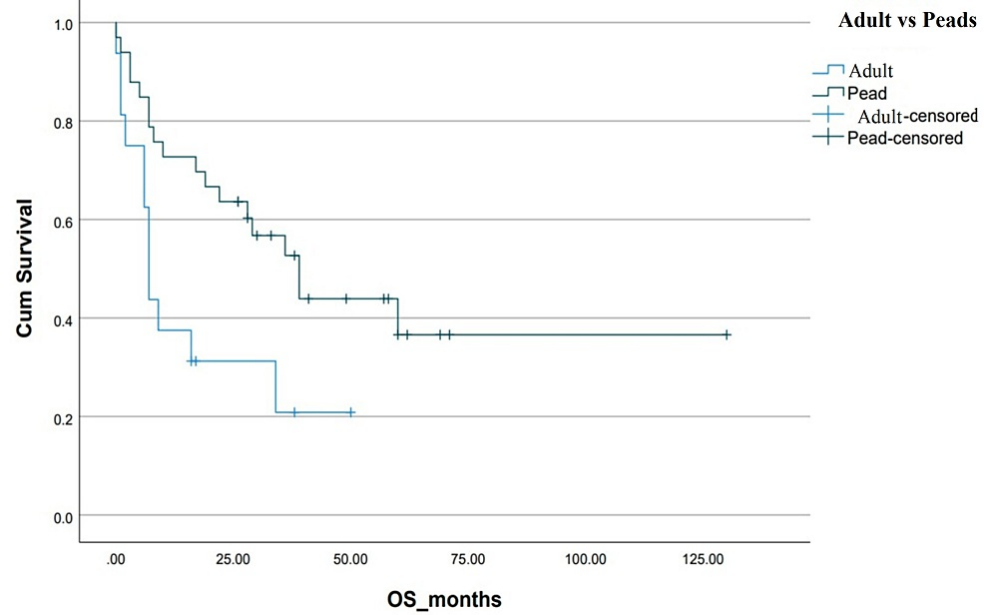
Kaplan-Meier curve depicting OS in relation to chemotherapy protocol. OS: overall survival; Pead: pediatric; Cum survival: cumulative survival

**Figure 6:**
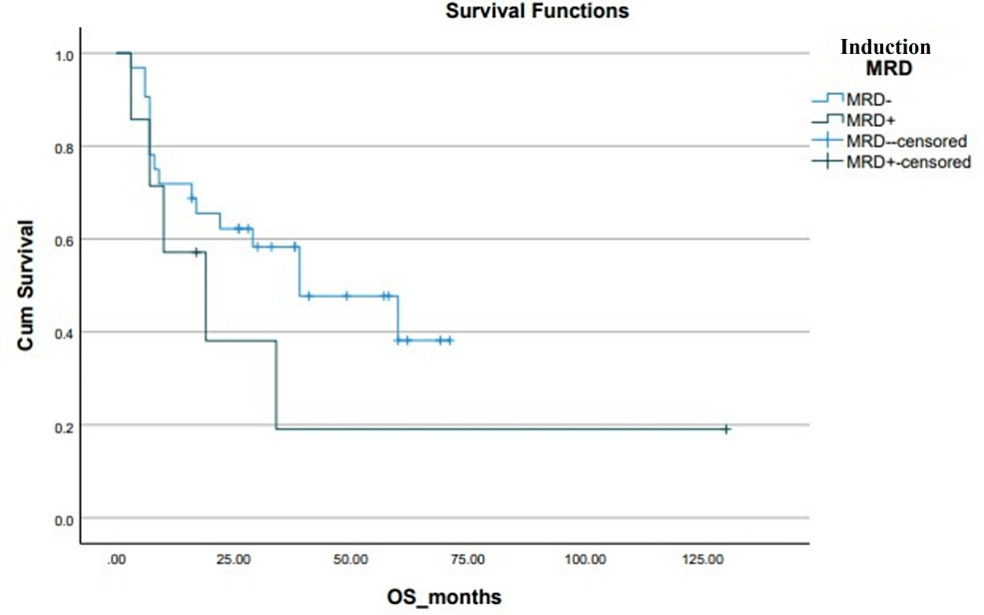
Kaplan-Meier curve depicting OS in relation to induction MRD. OS: overall survival; MRD: minimal residual disease; Cum survival: cumulative survival

**Figure 7:**
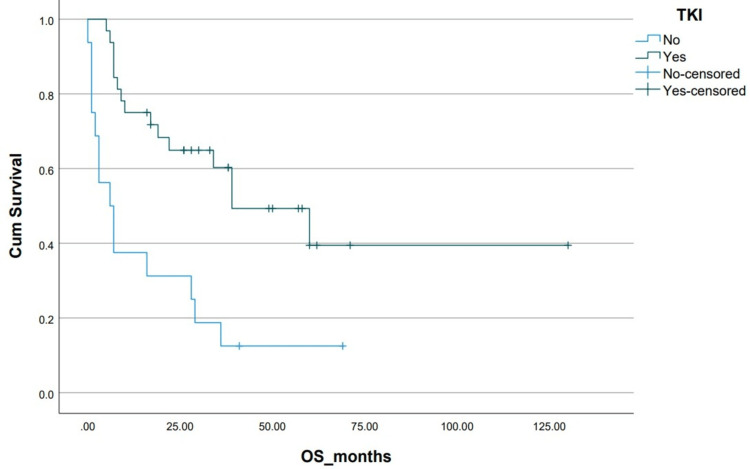
Kaplan-Meier curve depicting OS in relation to TKI use. OS: overall survival; TKI: tyrosine kinase inhibitor; Cum survival: cumulative survival

**Figure 8:**
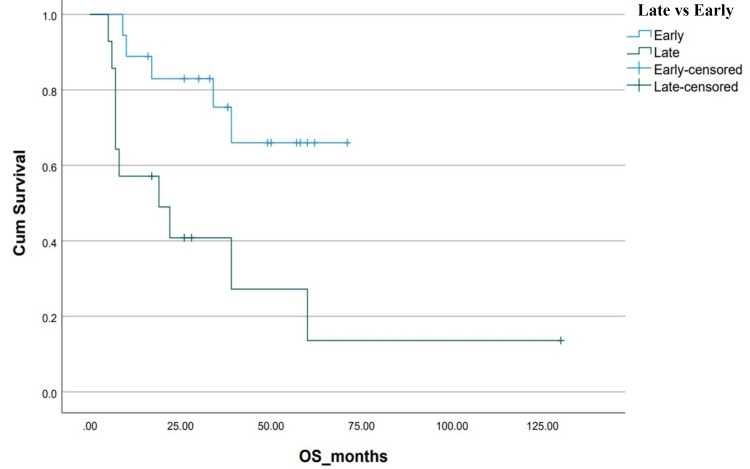
Kaplan-Meier curve depicting OS in relation to the timing of TKI. OS: overall survival; TKI: tyrosine kinase inhibitor; Cum survival: cumulative survival

**Figure 9:**
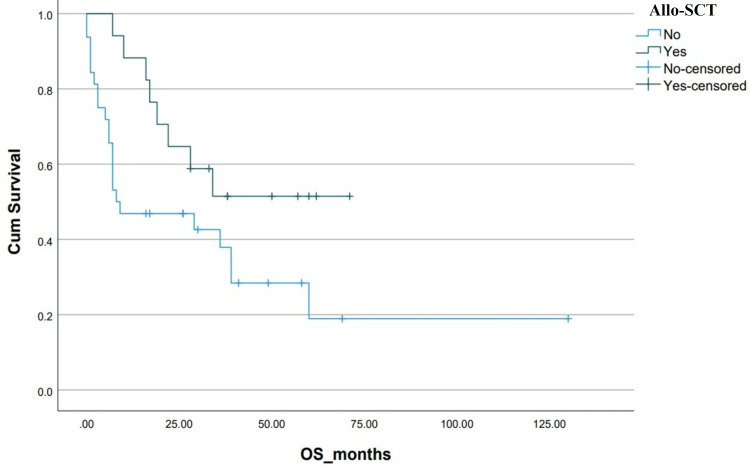
Kaplan-Meier curve depicting OS in relation to Allo-SCT. OS: overall survival; Allo-SCT: allogeneic stem cell transplant; Cum survival: cumulative survival

**Figure 10:**
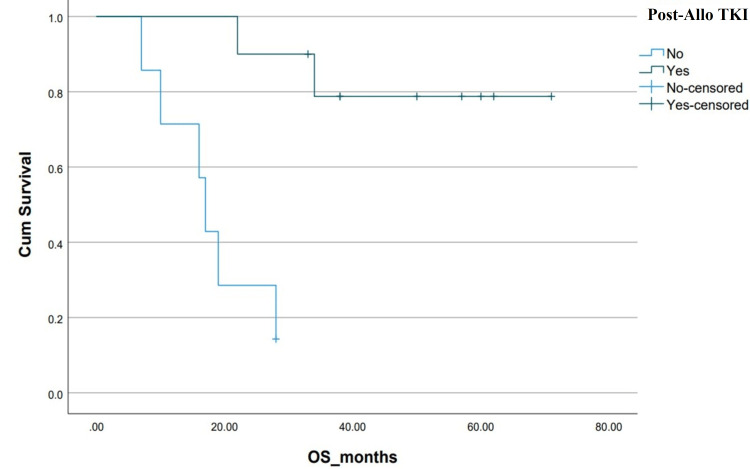
Kaplan-Meier curve depicting OS in relation to Post-Allo-SCT TKI. OS: overall survival; TKI: tyrosine kinase inhibitor; Allo-SCT: allogeneic stem cell transplant; Cum survival: cumulative survival

The estimated five-year survival, DFS and OS, of the entire cohort were 23% and 25%, respectively. The median OS in the Allo-SCT group was not reached vs 8±8.8 months (p=0.05) in the chemotherapy-only group. OS was found to be associated significantly with the presence of additional cytogenetic abnormalities, adult or pediatric-inspired chemotherapy protocols, the addition of TKIs during the induction phase, Allo-SCT and post-Allo-SCT use of TKIs (Table 2 and Figures 3-10).

**Table 2:** Factors determining OS and DFS. Allo-SCT: allogeneic stem cell transplant; DFS: disease-free survival; MRD: minimal residual disease; OS: overall survival; TKI: tyrosine kinase inhibitor; WBC: white blood cell; NR: not reached

Variables	Median OS (months)	p-Value	Median DFS (months)	p-Value
WBC count				
<30,000	34±8.90	0.79	27±10.34	0.59
>30,000	16±5.99		14±6.04	
Karyotyping				
t(9;22)	36±5.33	0.002	36±16.07	0.03
Additional	7±0.57		5±0.894	
Chemotherapy protocol				
Pediatric inspired	39±7.33	0.01	27±10.60	0.962
Adult	7±0.66		6±1.06	
Induction MRD				
MRD negative	39±15.32	0.25	37±14.85	0.459
MRD positive	19±19.41		18±8.04	
TKI addition				
Yes	39±12.78	<0.01	17±3.41	0.117
No	6±2.66		6±2.78	
TKI timing				
Early	NR	0.07	NR	0.191
Late	19±11.68		19±10.7	
Allo-SCT				
Yes	NR	0.05	37±13.1	0.892
No	8±8.8		8±6.2	
Post-Allo-SCT TKI				
Yes	NR	<0.01	NR	0.065
No	17±1.30		16±10.9	

## Discussion

The addition of TKIs to the chemotherapy backbone during induction has improved both hematological response as well as OS in Ph+ ALL in adults and children [8,9]. Considering the high induction mortality associated with chemotherapy, there is a paradigm shift towards more CNS-penetrant TKIs and chemo-free regimens like blinatumomab with improved treatment outcomes [10]. In a few instances, remissions have been deep and durable, thereby challenging and reducing the need for both chemotherapy and Allo-SCT in the first remission [11]. The treatment landscape and outcomes are less optimistic in resource-limited countries where CNS-penetrant TKIs like dasatinib and bispecific T-cell engagers like blinatumomab are not readily available.

Our patient population consisted of the AYA age subgroup, recognized as a unique population with specific characteristics and needs. Our AYA Ph+ ALL patients with high white blood count [12] and additional cytogenetics abnormalities on karyotyping had inferior survival outcomes similar to what is demonstrated in other age subgroups [13]. Patients were treated with either adult chemotherapy protocols (hyper-CVAD) or pediatric-inspired protocols like (BFM 2000 or UK-ALL regimens). These chemotherapy regimens resulted in a high induction mortality rate of 16% with Gram-negative rod sepsis and fungal pneumonia being the primary causes of death. Subset univariate analysis showed an improved OS for patients receiving pediatric-inspired regimens (39±7.33 vs 7±0.66 months, p=0.01), which is in line with established practice for Ph+ ALL AYA patients [14]. This high induction mortality does make a strong case for pursuing a more potent TKI plus less intensive or non-chemotherapeutic combinations at the outset of treatment.

Induction MRD assessment is a promising prognostic marker in the management of Ph+ ALL and MRD positivity is associated with a higher risk of relapse and mortality [15]. Our study demonstrated similar (39±15.32 vs 19±19.41 months, p=0.25) results, however, these were not statistically significant. This is likely due to MRD assessment using a flow cytometry-based technique with low sensitivity score of 10-2, in contrast to qualitative PCR-based testing of BCR-ABL transcripts, which have a higher sensitivity score of 10-5 [16].

The addition of TKIs to chemotherapy led to better OS (39±12.78 vs 6±2.66 months, p=0.07). Early incorporation of TKIs in the induction phase demonstrated even greater OS benefits (NR vs 19±11.68 months, p=0.05). However, as evident from the treatment flow chart, only 66% of the total population could afford TKIs (imatinib or nilotinib). None of the patients received third-generation TKIs which have higher central nervous system penetration [17]. TKIs, though highly effective, place a significant financial burden on patients in low-income countries and financing treatment is an ordeal shared by patients of CML in low-income countries [18]. As a consequence, most patients received imatinib at a dose of 400 mg once daily rather than a 600-800 mg dose. The generic availability of imatinib provides a more cost-effective option for our patient population [19].

Patients who received Allo-SCT had considerably improved OS in our patients (NR vs 8±8.8 months, p=0.01). Before the TKI era, Allo-SCT was considered the only curative treatment option available for patients with Ph+ ALL in CR1 with long-term survival of 40-50%, however, donor availability and treatment-related mortality (TRM) limit its feasibility [20,21]. The recent development of third-generation TKIs and blinatumomab have questioned the role of Allo-SCT in CR1.

Two recent meta-analyses demonstrate a survival advantage for Allo-SCT with the use of imatinib, but the advantage seems to disappear with newer TKIs due to their greater efficacy compared to imatinib, as well as the TRM rate of Allo-SCT [22,23]. Considering the financial burden of newer TKIs and novel therapies in resource-limited countries, Allo-SCT continues to be an attractive option for our patients when under treatment with curative intent. Relapse after Allo-SCT is a common reason for treatment failure and post-Allo-SCT administration of TKIs represents a concrete strategy to prevent Ph+ ALL recurrence. In our study, patients receiving TKIs post-Allo-SCT demonstrated improved OS (NR vs 17±1.30 months, p<0.01) [24,25]. In fact, the best estimated five-year OS of 80% was seen in the patient sub-group who received Allo-SCT followed by TKI maintenance, in our study.

The median DFS of 27 months (range: 0.9-53.1 months) and median OS of 29 months (range: 8.8-49.1 months), for our entire cohort, are less than those seen in other centers in South Asia with only around 25% of patients surviving long-term in our patient cohort [26]. Lack of standardized diagnostic facilities like MRD assessment using real-time PCR, non-availability of TKIs started early in induction, non-availability of third-generation TKIs, and lack of access to blinatumomab and Allo-SCT are critical factors that appear to contribute to the poor OS and DFS seen in our study. The difference in median PFS and OS of just two months suggests that most relapsed patients do not fare well in low-income countries. Due to lack of effective salvage therapies at time of relapse, they generally get referred to palliative services. There remains an unmet need for the improvement of diagnostic modalities and therapeutic intervention for Ph+ ALL AYA patients in low-income countries. As long as this need is not met, chemotherapy plus the early introduction of TKIs followed by Allo-SCT and post-Allo-SCT TKI maintenance seems to be the best hope of cure and long-term survival.

We recognize that a relatively small number of patients of this rare disease of AYA, MRD assessment without real-time PCR, limited access to TKIs, and the retrospective nature of the study are limitations of our study.

## Conclusions

Early addition of TKIs to pediatric-inspired chemotherapy protocols followed by Allo-SCT and post-Allo-SCT TKI maintenance in Ph+ ALL AYA patients results in improved OS. The treatment paradigm is shifting towards blinatumomab and third-generation TKIs, which show improved survival and durable remissions even without Allo-SCT. Lack of standardized diagnostic tools like MRD, TKI, and blinatumomab unavailability remains an issue in low-income countries due to the significant financial burden on the patients. Allo-SCT continues to be an attractive option in low-income countries providing an option for cure.
